# Supplementary material for: Human–robot interaction: predicting research agenda by long short-term memory
Source: PeerJ Comput Sci. 2024 Oct 25;10:e2335. doi: 10.7717/peerj-cs.2335 (PMC11623198; doi:10.7717/peerj-cs.2335)
Supplement: Supplemental Information 1 [file peerj-cs-10-2335-s001.docx]

Dear Editors,

In our bibliometric analysis study, we have used VantagePoint software to perform text mining to identify patterns and trends in the literature related to human-robot interaction. The results obtained have been significant and relevant to our work.

However, we consider it important to note that the results obtained with VantagePoint can be reproduced using free tools available on the market. This alternative approach may be relevant to our fellow researchers seeking to replicate or expand our study without incurring additional costs.

For example, the use of programming languages such as R and Python, along with specialized libraries, provides robust capabilities for bibliometric data analysis and visualization. These tools are widely used in the scientific and academic community, and offer a viable option for those who wish to perform detailed analyses without relying on proprietary software.

In addition, online tools such as Voyant Tools offer specific functionality for text analysis, which can be relevant for certain aspects of bibliometric analysis, such as identifying trends and exploring relationships between terms.

If we delve deeper into Python, it is important to note that the same analyses can be reproduced using its specialized text mining libraries.

Python offers a robust ecosystem of natural language processing (NLP) tools that enable a wide range of text mining tasks to be performed. For example, libraries such as NLTK (Natural Language Toolkit), spaCy and scikit-learn provide tools for tokenization, parsing, feature extraction, text classification and much more.

In addition, there are specific libraries for bibliometric analysis, such as bibliometrix, which can be used in combination with Python NLP tools to perform detailed analyses of the scientific literature. These libraries enable tasks such as calculating bibliometric indicators, visualizing co-authorship networks, and identifying emerging themes in the literature.

The advantage of using Python lies in its flexibility and its large community of developers, who constantly contribute new tools and techniques for text analysis. In addition, Python is a free and open source programming language, making it accessible to researchers worldwide.

In summary, while VantagePoint offers a convenient and specialized solution for text analysis, the same analyses can be reproduced using Python and its text mining libraries, providing researchers with a free and versatile alternative for performing bibliometric analyses.

We remain at your disposal to provide further details on our methodology and results, as well as to discuss any additional aspects related to this topic.

Yours sincerely,

Jon Borregan.
